# Pregnancy in Rudimentary Horn

**DOI:** 10.7759/cureus.48015

**Published:** 2023-10-31

**Authors:** Gunjan Gunjan, Punit Hans

**Affiliations:** 1 Obstetrics and Gynecology, Patna Medical College, Patna, IND; 2 Obstetrics and Gynecology, Nalanda Medical College and Hospital, Patna, IND

**Keywords:** mullerian defect, emergency laprotomy, cornual pregnancy, rudimentary horn, ectopic pregnancy

## Abstract

Pregnancy in rudimentary horn is an uncommon presentation of an ectopic pregnancy. It needs a very high degree of suspicion for diagnosis and the diagnosis becomes difficult in cases with previous vaginal deliveries. A 25-year-old female patient with two spontaneous vaginal deliveries and a history of spontaneous abortion at five months visited the obstetric emergency department with a history of five months of amenorrhea with pain abdomen and breathlessness for one day. On abdominal examination abdomen was distended, and rigid, and tenderness was present. Paracentesis was done where blood was present. On bimanual examination, cervical motion tenderness was present, and left-sided fornices fullness was present. The patient was admitted, and her sonography was done for suspicion of a ruptured uterus or ectopic pregnancy. The sonography report showed a bulky uterus with decidual reactions and a well-defined pregnancy of 21 weeks and 6 days in the right adnexal region with hemoperitoneum suggesting suspicion of ruptured ectopic pregnancy. After initial treatment and arrangement of two units of packed red blood cells after proper grouping and cross-matching for the patient, laparotomy was done. At the time of surgery, there was a right-sided rupture of non-communicating rudimentary horn pregnancy with a unicornuate uterus. A dead fetus of 600 grams lies in the peritoneal cavity with two liters of hemoperitoneum. Timely diagnosis and laparotomy saved the life of the patient.

## Introduction

Cornual pregnancy is 2%-4% of all ectopic pregnancy [1]. A unicornuate uterus accounts for 2.4%-13% of all Mullerian anomalies [2]. 72-85% of rudimentary horns are non-communicating with the cavity [3]. Unicornuate uterus with rudimentary horns may be associated with complications like infertility, endometriosis, hematometra, and urinary tract abnormalities. Its most dangerous and life-threatening complication is rupture. The mortality rate due to cornual pregnancy is 2-5 times higher than other ectopic pregnancies as they are mostly misdiagnosed. Early diagnosis and treatment are important as the risk of rupture increases beyond 12 weeks.

## Case presentation

A 25-year-old female patient with two spontaneous vaginal delivery and a history of spontaneous abortion at five months visited the obstetric emergency department with a history of five months of amenorrhea with pain abdomen for one day which was aggravated by postural changes. She also complained of giddiness and breathlessness for which she took treatment in a private hospital but as her condition deteriorated, she was referred to a higher center. The patient had taken an antenatal checkup for this pregnancy and had taken iron folic acid, calcium, and one dose of tetanus toxoid. On examination, the patient was severely pale, ill-looking, and anxious. Her pulse was 160 bpm and her BP was 96/60 mm of Hg. On abdominal examination, the abdomen was distended with guarding and rigidity with generalized tenderness. Paracentesis was done where blood was present. On bimanual examination, cervical motion tenderness was present, and the left-sided fornix fullness was present.

**Figure 1:**
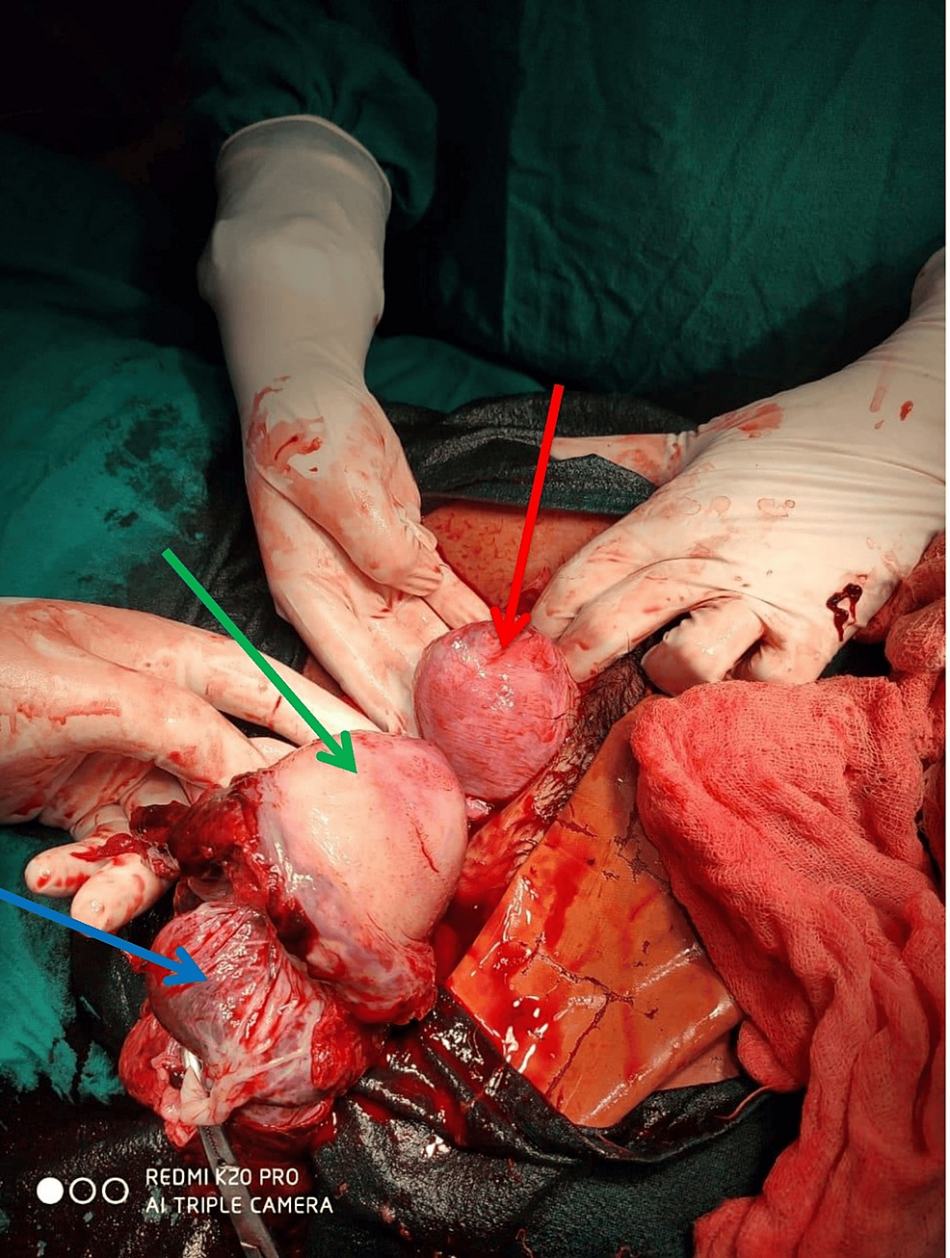
Ruptured accessory horn with placenta attached to it The green arrow shows the ruptured accessory horn of the uterus, the blue arrow shows the placenta attached to the accessory horn, and the red arrow shows the uterus.

**Figure 2:**
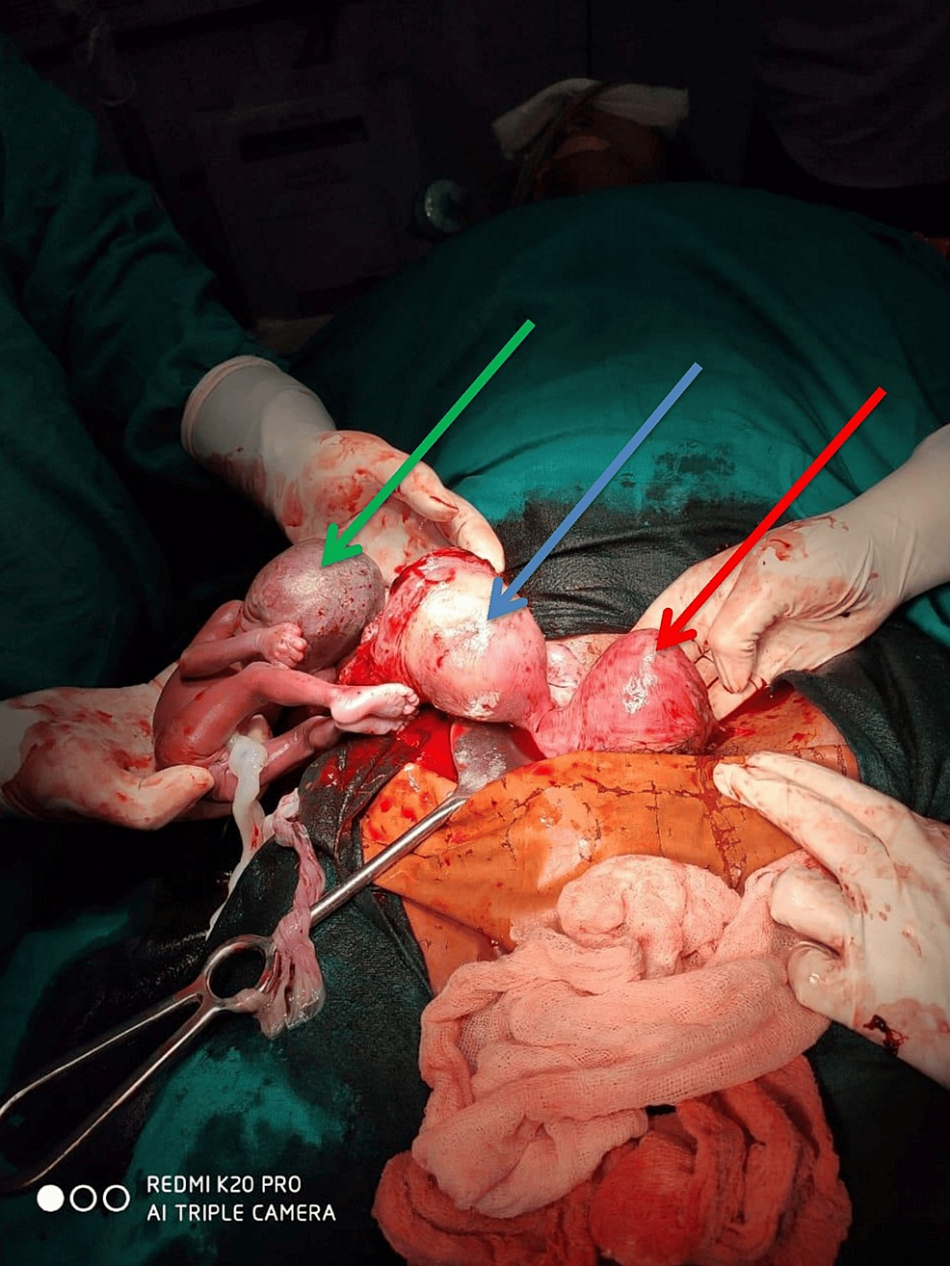
Ruptured accessory horn of the uterus The green arrow shows fetus that was lying in the peritoneal cavity, the blue arrow shows the ruptured accessory horn of the uterus, and the red arrow shows the uterus.

The patient was admitted, and her sonography was done which showed a well-defined pregnancy of 21 weeks 6 days in the right adnexal region with suspicion of rupture ectopic with a moderate amount of intraperitoneal collection. Her blood report showed a low red blood cell count, and her hemoglobin was 6.8g/dL. After initial treatment and arrangement of two units of packed red blood cells with proper grouping and cross-matching for the patient and two units of fresh frozen plasma laparotomy was done. Two liters of blood were removed from the abdominal cavity. There was a rupture of the right rudimentary non-communicating horn of unicornuate uterus (Figure 1). A dead fetus of 600 grams was lying in the peritoneal cavity with placenta attached to the ruptured accessory horn of the uterus as shown in Figures 1, 2, respectively. The ruptured right accessory horn was removed. The ovary was healthy and preserved and a tubectomy was done. The post-operative period was uneventful. The patient was later investigated for urinary tract anomalies and was found to have an ectopic malrotated right kidney.

## Discussion

Ectopic pregnancy is an extrauterine pregnancy where most ectopic pregnancy resides in the fallopian tube. There are other possible sites like cornual, intramural, cervical, cesarean scar, ovarian, or abdominal. Ectopic pregnancy generally manifests at six to eight weeks of gestation, but it may occur late when ectopic is located at other sites [4]. In this case, the patient presents with ruptured ectopic (accessory horn) at five months of pregnancy. The patient had her antenatal care as a normal intrauterine pregnancy till five months, even the previous ultrasonography reports could not detect an early rudimentary horn pregnancy in this case [5]. The onset of pain and abdominal distension led to confusion about the ruptured uterus as the patient was multigravida with two spontaneous vaginal delivery [6]. Unfortunately, the patient landed in a hemodynamically unstable condition where laparotomy was performed [7]. The patient was ligated as her family was completed. After her postoperative period patient was further evaluated where ultrasound and MRI showed an ectopic malrotated right kidney [8].

## Conclusions

Uncommon and vague symptoms of cornual ectopic pregnancy misleads clinician which results in fatal complications. As these pregnancies have high risk of rupture due to delayed diagnosis the presentation is late and the size of ectopic is bigger. This case report study suggests to explore all the possible sites of ectopic pregnancy and even in case of slightest suspicion immediate intervention is done to prevent rupture. Timely resustication and blood transfusion are needed to save the patient. So detection of the condition before pregnancy or before rupture is important .Despite taking proper antenatal check up the diagnosis of rudimentary horn pregnancy was missed .The early weeks sonography could not differentiate in an intrauterine pregnancy and a rudimentary horn pregnancy. Precious time gets lost due delayed diagnosis or misdiagnosis and the patient 's condition deteriorates as it happenened in this case .Increased awareness of this condition is important as the patient generally lands in a serious condition and time gets wasted in diagnosis and referral to higher centres.
